# *Helicobacter pylori* Infection in Thailand: A Nationwide Study of the CagA Phenotype

**DOI:** 10.1371/journal.pone.0136775

**Published:** 2015-09-10

**Authors:** Tomohisa Uchida, Muhammad Miftahussurur, Rapat Pittayanon, Ratha-korn Vilaichone, Naruemon Wisedopas, Thawee Ratanachu-ek, Tetsuko Kishida, Masatsugu Moriyama, Yoshio Yamaoka, Varocha Mahachai

**Affiliations:** 1 Department of Molecular Pathology, Oita University Faculty of Medicine, Hasama-machi, Yufu-City, Oita, Japan; 2 Department of Environmental and Preventive Medicine, Oita University Faculty of Medicine, Hasama-machi, Yufu-City, Oita, Japan; 3 Institute of Tropical Disease, Airlangga University, Surabaya, Indonesia; 4 Division of Gastroenterology, Chulalongkorn University, Bangkok, Thailand; 5 Gastroenterology Unit, Thammasat University Hospital, Pathumthani, Thailand; 6 Department of Pathology, Chulalongkorn University Hospital, Bangkok, Thailand; 7 Department of Surgery, Rajavithi Hospital, Bangkok, Thailand; 8 Department of Forensic Medicine, Oita University Faculty of Medicine, Hasama-machi, Yufu-City, Oita, Japan; 9 Department of Molecular Pathology, Oita University Faculty of Medicine, Hasama-machi, Yufu-City, Oita, Japan; 10 Department of Medicine-Gastroenterology, Baylor College of Medicine and Michael E. DeBakey Veterans Affairs Medical Center, Houston, Texas, United States of America; 11 Gastrointestinal and Liver Center, Bangkok Medical Center, Bangkok, Thailand; University of Hyderabad, INDIA

## Abstract

**Background:**

The risk to develop gastric cancer in Thailand is relatively low among Asian countries. In addition, the age-standardized incidence rate (ASR) of gastric cancer in Thailand varies with geographical distribution; the ASR in the North region is 3.5 times higher than that in the South region. We hypothesized that the prevalence of *H*. *pylori* infection and diversity of CagA phenotype contributes to the variety of gastric cancer risk in various regions of Thailand.

**Methods:**

We conducted a nationwide survey within Thailand. We determined *H*. *pylori* infection prevalence by detecting *H*. *pylori*, using histochemical and immunohistochemical methods. The anti-CagA antibody and anti-East-Asian type CagA antibody (α-EAS Ab), which showed high accuracy in several East Asian countries, were used to determine CagA phenotype.

**Results:**

Among 1,546 patients from four regions, including 17 provinces, the overall prevalence of *H*. *pylori* infection was 45.9% (710/1,546). Mirroring the prevalence of *H*. *pylori* infection, histological scores were the lowest in the South region. Of the 710 *H*. *pylori-*positive patients, 93.2% (662) were immunoreactive with the anti-CagA antibody. CagA-negative strain prevalence in the South region was significantly higher than that in other regions (17.9%; 5/28; *p* < 0.05). Overall, only 77 patients (11.6%) were immunoreactive with the α-EAS Ab. There were no differences in the α-EAS Ab immunoreactive rate across geographical regions.

**Conclusions:**

This is the first study using immunohistochemistry to confirm *H*. *pylori* infections across different regions in Thailand. The prevalence of East-Asian type CagA *H*. *pylori* in Thailand was low. The low incidence of gastric cancer in Thailand may be attributed to the low prevalence of precancerous lesions. The low incidence of gastric cancer in the South region might be associated with the lower prevalence of *H*. *pylori* infection, precancerous lesions, and CagA-positive *H*. *pylori* strains, compared with that in the other regions.

## Introduction

*Helicobacter pylori* is a spiral-shaped, gram-negative bacterium that chronically colonizes the human stomach and is a causative agent of various gastroduodenal diseases, including gastritis, peptic ulcers, gastric cancer (GC), and mucosa-associated lymphoid tissue lymphoma [1]. Although *H*. *pylori* infection is a major factor in the development of GC [2], the differences in *H*. *pylori* infection rates are insufficient to explain the differences in the incidence of GC worldwide [3]. In Thailand, the reported *H*. *pylori* infection rate ranges from 54.1% to 76.1% [4]; however the age-standardized incidence rate (ASR) of GC was reported to be 3.1/100,000, which is relatively low among Asian countries (available from the International Agency for Research on Cancer; GLOBOCAN2012, http://globocan.iarc.fr/) [5].

Interestingly, the ASR of GC in Thailand varied based on geographical distribution. The North region has the highest incidence rate (6.45 for men and 4.35 for women), whereas the South region has the lowest rate (1.9 for men and 1.4 for women). A previous study attributed differences in incidence of GC to environmental factors including consumption of salt, nitrates, and vegetables [6]. However, in addition to host and environmental factors, the difference in the incidence of GC, irrespective of *H*. *pylori* infection rate, can be explained by differences in the virulence factors of *H*. *pylori* [7].

*cagA*, which encodes a highly immunogenic protein (CagA), is the most extensively studied *H*. *pylori* virulence factor [8]. There are two types of clinical *H*. *pylori* isolates: CagA-producing (CagA-positive) strains and CagA non-producing (CagA-negative) strains. CagA was typed on the basis of the sequences of the 3’-region of the *cagA* gene, which contains the Glu-Pro-Ile-Tyr-Ala (EPIYA) motif [9]. Sequences have been annotated according to the segments (20–50 amino acids) flanking the EPIYA motifs (i.e., segments EPIYA-A, B, C or D). The East-Asian type CagA, containing the EPIYA-D segment, exhibits a stronger binding affinity for Src homology 2 (SHP-2) and a greater ability to induce morphological changes in epithelial cells than does the Western type CagA, which contains the EPIYA-C segment [10]. As a result, the East-Asian type CagA is considered to be more toxic than its Western homologues and more strongly associated with severe clinical outcomes, including gastric cancer [11].

Although several histochemical stains used for the detection of *H*. *pylori* in gastric biopsies could enhance visualization of the organism compared to that achieved with routine hematoxylin and eosin staining [12], several studies have shown that, compared to histochemical stains, immunohistochemical (IHC) staining with specific *H*. *pylori* antibodies has the highest sensitivity and specificity, and results in greater inter-observer agreement [13]. Recently, we also successfully generated an anti-East-Asian type CagA-specific antibody (α-EAS Ab), which was immunoreactive only with the East-Asian type CagA and not with the Western type CagA [14]. We have also shown that the α-EAS Ab is a useful tool for typing CagA immunohistochemically in Japan [15] and in Vietnam and Thailand [16], with a sensitivity, specificity, and accuracy of 93.2%, 72.7%, and 91.6%, respectively, in Vietnam and 96.7%, 97.9%, and 97.1%, respectively, in Thailand. In this study, we used IHC to confirm *H*. *pylori* infection by histopathology in a large number of samples obtained from several regions in Thailand. Furthermore, we also identified CagA phenotypes and analyzed the influence of *H*. *pylori* CagA diversity on gastric mucosal status in Thailand.

## Material and Methods

### Study population

**Fig 1:**
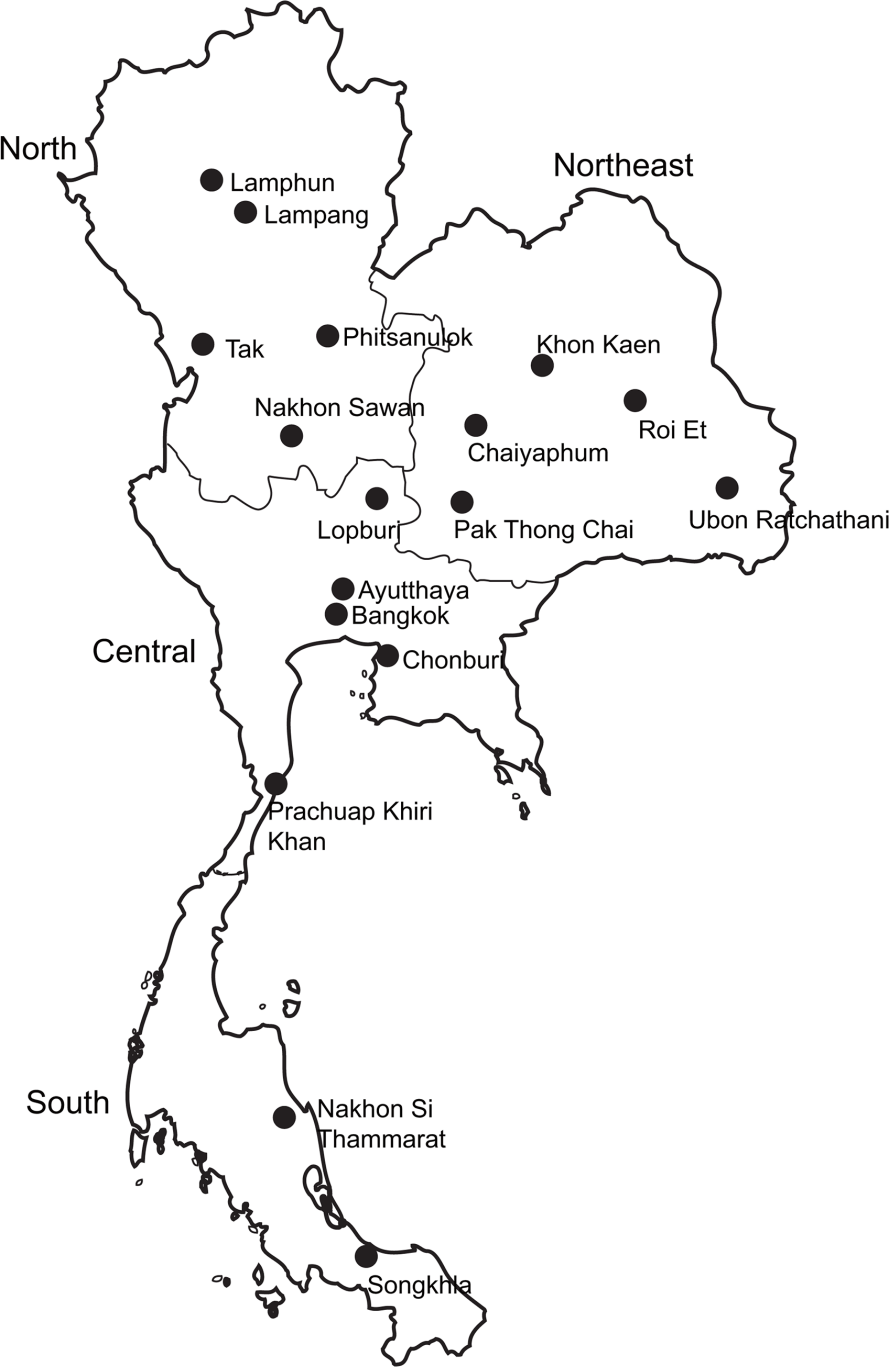
A nationwide community-based endoscopic survey of different regions of Thailand. Consecutive patients (n = 1,546) with dyspepsia were enrolled from the North, Northeast, Central, and South regions.

From February 2008 to May 2013, we conducted a nationwide community-based endoscopic survey in different regions of Thailand (Fig 1). Patients aged ≤18 years, and those who had received *H*. *pylori* eradication therapy or had received proton pump inhibitors (PPI), H2-receptor antagonists, bismuth, antibiotics, and nonsteroidal anti-inflammatory drugs in the month prior to this study were excluded [17].

Experienced endoscopists collected two gastric biopsy specimens during each endoscopy session: one sample from the lesser curvature of the antrum, at approximately 3 cm from the pyloric ring, and one sample from the greater curvature of the corpus. Written informed consent was obtained from all participants, and the study protocol was approved by the Ethics and Research Committee of Chulalongkorn University Faculty of Medicine (Bangkok, Thailand) and Oita University Faculty of Medicine (Yufu, Japan).

All biopsy materials for histological testing were fixed in 10% buffered formalin and embedded in paraffin. Serial sections were stained with hematoxylin and eosin, as well as May–Giemsa stain. Gastric mucosa were evaluated by an experienced pathologist (TU) based on the updated Sydney system [18].

IHC was performed as previously described [15]. Briefly, after antigen retrieval and inactivation of endogenous peroxidase activity, tissue sections were incubated overnight at 4°C with anti-*H*. *pylori* antibody (DAKO, Denmark), anti-CagA antibody (b-300 Santa Cruz, USA), or α-EAS Ab diluted 1:2000 with the diluting solution (DAKO, Denmark). After washing, the sections were incubated with biotinylated goat anti-rabbit or anti-rat IgG (Nichirei Co., Japan), which was followed by incubation with a solution of avidin-conjugated horseradish peroxidase (Vectastain Elite ABC kit; Vector Laboratories Inc., Burlingame, CA, USA). Peroxidase activity was detected using H_2_O_2_/diaminobenzidine substrate solution. The bacterial load was classified into four grades: 0, ‘None’; 1, ‘mild’; 2, ‘moderate’; and 3, ‘marked’ by specimens stained with May-Giemsa [18]. Positive immunoreactivity was judged as follows: *H*. *pylori* were identified by May-Giemsa staining and positively immunostained with anti-*H*. *pylori* antibody, with bacterial loads greater than or equal to grade 1 being considered positive for *H*. *pylori*.

### Determination of gastritis stage

Degree of inflammation, neutrophil activity, atrophy, intestinal metaplasia, and bacterial density were classified into four grades according to the updated Sydney system: 0, ‘normal’; 1, ‘mild’; 2, ‘moderate’; and 3, ‘marked’ [18]. Samples classified as grade 1 or more for atrophy were considered atrophy-positive [19]. An inflammation score equal to or greater than 1 was considered as chronic gastritis, while active gastritis was defined as a neutrophil score equal to or greater than 1. Active gastritis was classified based the topographical distribution of activity: antral-predominant gastritis (antrum > corpus) or corpus-predominant gastritis (corpus > antrum) if there was ≥1-point difference between the scores for activity, or as pangastritis if the difference in the grade of activity was <1-point between the corpus and antrum [20]. In addition, gastritis stage was assessed based on topographic locations (antrum and corpus), according to the Operative Link on Gastritis Assessment (OLGA) system [21].

### Statistical analysis

Data were analyzed using SPSS, version 19 (SPSS Inc., Chicago, IL, USA). Discrete variables were tested using the chi-square test; continuous variables were tested using Mann-Whitney *U*. Somers’ D coefficients (r) were determined to evaluate the association between the intensity of *H*. *pylori* colonization and histological scores. A multivariate logistic regression model was used to calculate the odds ratios (OR) of the clinical outcomes by considering age, sex, *H*. *pylori* infection, and gastritis type. All determinants with *p*-values < 0.10 were included in the full model of logistic regression, and the model was reduced by excluding variables with P-values > 0.10. The OR and 95% confidence interval (CI) were used to estimate the risk. A *p*-value ≤ 0.05 was considered statistically significant.

## Results

### *H*. *pylori*-related gastritis in Thailand

In total, 1,546 consecutive patients (1,065 women and 481 men; mean age, 50.5 ± 12.2 years) with dyspepsia were enrolled from four regions, including 17 provinces (Fig 1). The patients were from the North region: Nakhon Sawan, Tak, Lampang, Phitsanulok, and Lamphun (n = 482); the Northeast region: Khon kaen, Chaiyaphum, Ubon Rachathanee, Pak Thong Chai, and Roi Et (n = 541); Central region: Lopburi, Bangkok, Prachuap Khiri Khan, Ayutthaya, and Chonburi (n = 328); and the South region: Nakhon Si Thammarat and Songkhla (n = 195) (Table 1). The total study population consisted of 62 patients aged ≤29 years, 221 patients aged 30–39 years, 448 patients aged 40–49 years, 460 patients aged 50–59 years, and 355 patients aged ≥60 years.

**Table 1:** The prevalence of *H*. *pylori* infection, CagA-positive and EAS-positive in Thailand.

District	Province	Cases (1546)	Male	*H*. *pylori-positive*	%	CagA-positive	%	α-EAS-positive	%
**North**	Nakhon Sawan	139	39	56	40.3	53	94.6	3	5.7
	Tak	129	45	53	41.1	50	94.3	3	6.0
	Lampang	112	30	66	58.9	65	98.5	6	9.2
	Phitsanulok	89	26	44	49.4	41	93.2	4	9.8
	Lamphun	13	4	7	53.8	6	85.7	0	0.0
	**Total**	**482**	**144**	**226**	**46.9**	**215**	**95.1**	**16**	**7.4**
**Northeast**	Khon Kaen	135	45	82	60.7	75	91.5	11	14.7
	Chaiyaphum	122	34	72	59.0	67	93.1	7	10.4
	Ubon Rachathanee	120	36	81	67.5	77	95.1	13	16.9
	Pak Thong Chai	90	31	48	53.3	45	93.8	6	13.3
	Roi Et	74	27	45	60.8	44	97.8	4	9.1
	**Total**	**541**	**173**	**328**	**60.6**	**308**	**93.9**	**41**	**13.3**
**Central**	Lopburi	116	34	48	41.4	47	97.9	1	2.1
	Bangkok	99	35	30	30.3	26	86.7	5	19.2
	Prachuap Khiri Khan	60	20	24	40.0	21	87.5	5	23.8
	Ayutthaya	42	9	23	54.8	19	82.6	5	26.3
	Chonburi	11	2	3	27.3	3	100.0	1	33.3
	**Total**	**328**	**100**	**128**	**39.0**	**116**	**90.6**	**17**	**14.7**
**South**	Nakhon Si Thammarat	98	35	11	11.2	10	90.9	2	20.0
	Songkhla	97	29	17	17.5	13	76.5	1	7.7
	**Total**	**195**	**64**	**28**	**14.4**	**23**	**82.1**	**3**	**13.0**

**Fig 2:**
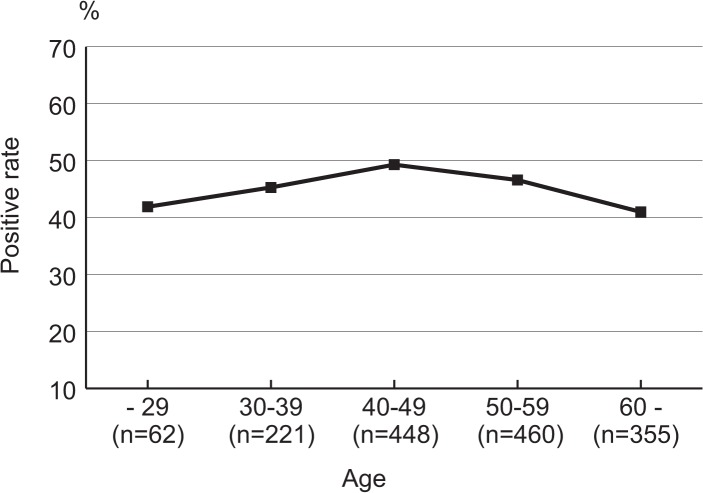
Prevalence of *H*. *pylori* infection in Thailand according to the age group. Subjects with positive histology and immunohistochemistry results were considered positive for *H*. *pylori* infection.

Hematoxylin and eosin staining, with May–Giemsa confirmation by IHC, revealed an overall prevalence of *H*. *pylori* infection of 45.9% (710/1,546) in Thailand. Men showed higher risk for *H*. *pylori* infection (OR = 1.67) than did women. The infection rate according to the age group was 43.5% (27/62) for patients aged ≤29 years, 45.7% (101/221) for patients aged 30–39 years, 49.3% (221/448) for patients aged 40–49 years, 47.0% (216/460) for patients aged 50–59 years, and 41.0% (145/355) for patients aged ≥60 years (Fig 2). The prevalence of *H*. *pylori* infection in the South region of Thailand (14.4%) was the lowest, compared with the Northeast (60.6%), North (46.9%), and Central (39.0%) regions (all *p* < 0.001; Table 1). Even after adjustment for age and sex in a multivariate analysis, the Central, North, and Northeast regions (OR = 3.90, OR = 5.43, and OR = 9.52, respectively) were at a significantly higher risk for *H*. *pylori* infection than was the South region.

### Gastric mucosa status in Thailand

**Fig 3:**
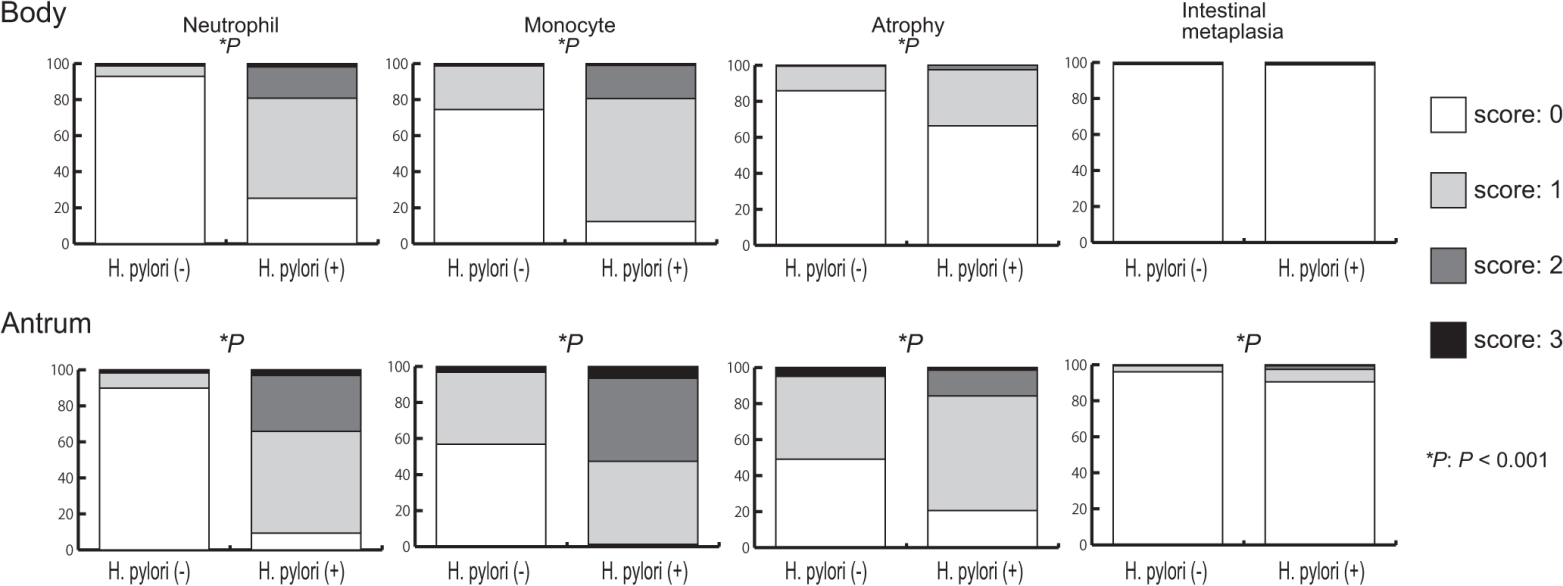
Histological scores according to *H*. *pylori* infection status. Activity, inflammation, and atrophy in both the antrum and corpus, and intestinal metaplasia only in the antrum were significantly higher in patients positive for *H*. *pylori* than in patients negative for *H*. *pylori*.

Histological scores according to *H*. *pylori* infection status are shown in Fig 3. Activity, inflammation, and atrophy levels in both the antrum and corpus were significantly higher in *H*. *pylori*-positive patients than in *H*. *pylori*-negative patients (all *p* < 0.001). However, intestinal metaplasia in the antrum, but not in the corpus, was significantly higher in *H*. *pylori*-positive patients than in *H*. *pylori*-negative patients (*p* < 0.001), probably due to the low incidence of intestinal metaplasia observed overall (nine cases in each group). The intensity of *H*. *pylori* colonization correlated with the levels of activity, inflammation, atrophy, and intestinal metaplasia in the antrum (r = 0.70, 0.63, 0.29, 0.03, all *p* < 0.01, respectively), and in the corpus (r = 0.65, 0.60, 0.26, 0.05, all *p* < 0.001, respectively). A patient infected with *H*. *pylori* was at a significantly high risk for gastritis (antral-predominant + corpus predominant and pan-gastritis) when adjusted for age and sex (OR = 43.47; 95% CI, 26.68–70.83).

According to the OLGA system, 34.0% (526/1,546) had stage 0. Stage I was found in 55.7% (861/1,546) of patients, 8.6% (133/1,546) had stage II, 1.5% had stage III (23/1,546), and only 0.2% (3/1,546) had stage IV. The clinical outcomes among *H*. *pylori*-infected patients in Thailand, according to the classification of gastritis, are shown in Table 2. Among the 710 patients infected with *H*. *pylori*, 99.6% (707/710) and 95.8% (680/710) had chronic and active gastritis, respectively. The highest rate of pangastritis was noted among patients that had active gastritis (304/680, 44.7%), followed by those with antrum-predominant gastritis (281/680, 41.3%), corpus-predominant gastritis (95/680, 14.0%), and intestinal metaplasia in the antrum and corpus (71/680, 10.4%).

**Table 2:** Clinical outcomes among *H*. *pylori*-infected patients in Thailand.

Disease	n = 710	%
Chronic gastritis	707	99.6%
Active gastritis	680	95.8%
Antrum-predominant gastritis	281	41.3%
Corpus-predominant gastritis	95	14.0%
Pangastritis	304	44.7%
Intestinal metaplasia	71	10.4%

### Comparison of gastric mucosa status for each region in Thailand

**Fig 4:**
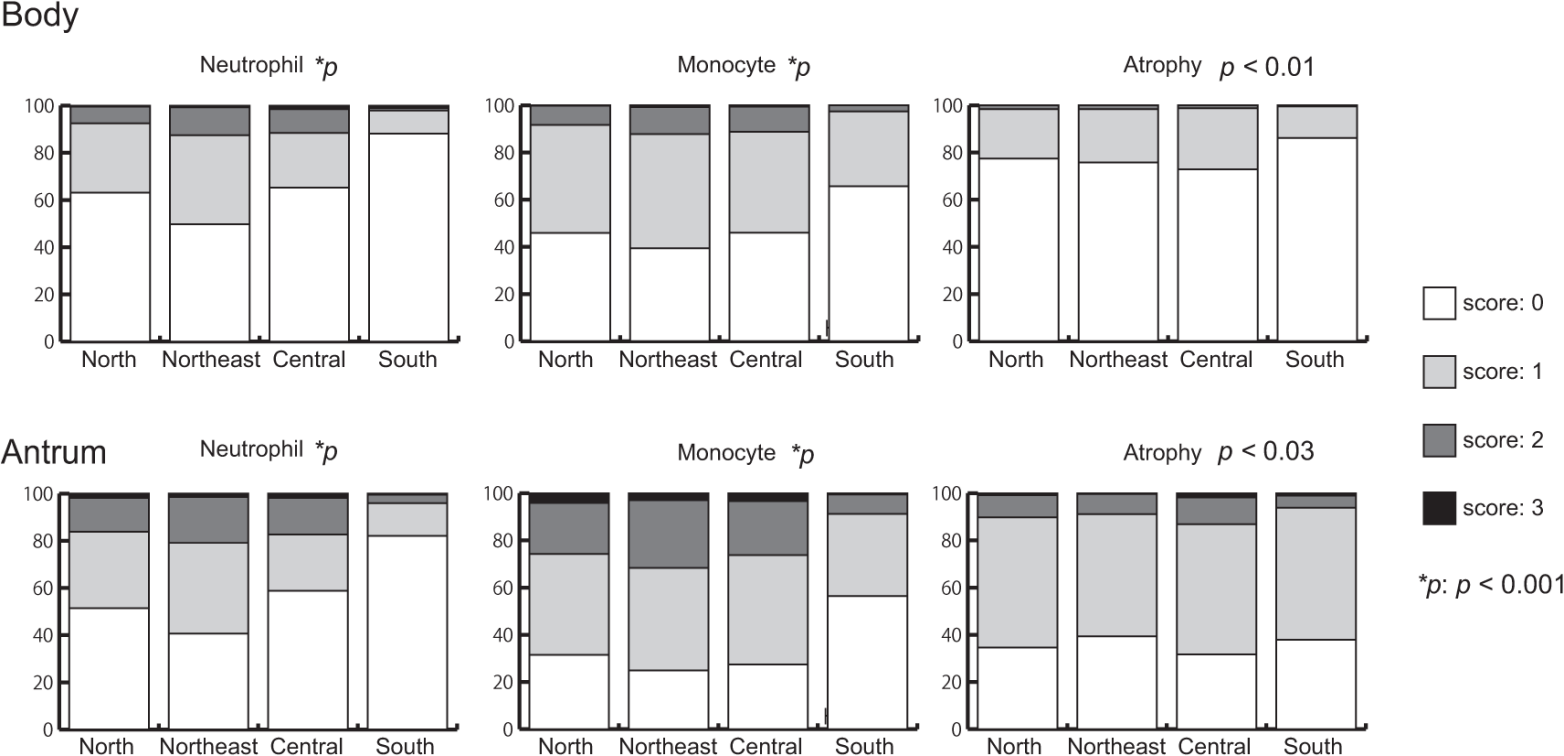
Comparison of gastric mucosa status for each region in Thailand. Of the four regions, patients from the Northeast region had the highest level of activity (active gastritis) and inflammation in both the antrum and corpus. Patients from the South region had the lowest atrophy in the corpus.

Of the four regions, patients from the Northeast region had the highest levels of activity and inflammation in both the antrum and corpus (all *p* < 0.001). In addition, patients from the North and Central regions had higher levels of activity and inflammation in both the antrum and corpus than did patients from the South region (*p* < 0.001). Although only patients from the Central region had a higher score for atrophy in the antrum than patients from the South region (*p* < 0.03), patients from the South region had the lowest atrophy in the corpus (*p* < 0.01; Fig 4). There were no significant differences in histological scores between patients from the Central and North regions (*p* > 0.05).

In concordance with gastric mucosa status, determined by the updated-Sydney system, gastritis status based on the topographical distribution of activity for each geographical region in Thailand revealed that the South region had the lowest gastritis rate: 9.2% (18/195) with antral-predominant gastritis, 2.6% (5/195) with corpus predominant gastritis, and 8.2% (16/195) with pangastritis. These rates were significantly different from those in the Central region (18.9%, 9.1%, and 18.0%), North region (24.7%, 7.7%, and 21.2%), and Northeast region (25.5%, 10.2%, and 28.0%) for antral-predominant gastritis, corpus predominant gastritis, and pangastritis, respectively (all *p* < 0.001). By multivariate analysis, the South region was found to have a lower risk for gastritis than the Central, North, and Northeast regions (OR 8.35, 3.85, and 13.88, respectively). However, there was no difference in gastritis status between regions after adjusting for *H*. *pylori* infection (all *p* > 0.05).

### Diversity of CagA in Thailand

**Fig 5:**
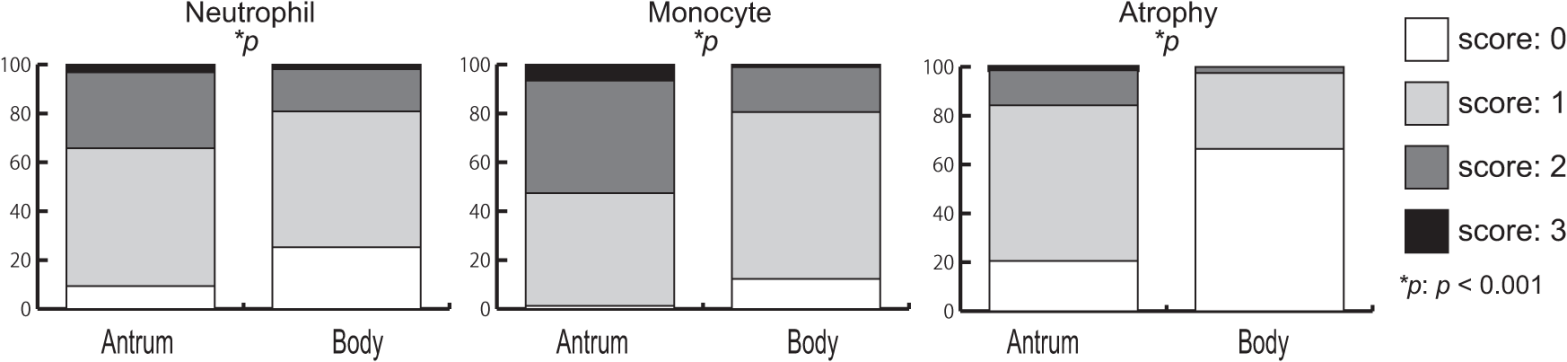
Patients infected with CagA-positive *H*. *pylori* show higher histological scores (activity, inflammation, and atrophy) than do patients infected with CagA-negative *H*. *pylori*.

Of the 710 *H*. *pylori-*positive patients enrolled in this study, 662 (93.2%) were immunoreactive for the anti-CagA antibody. Therefore, the remaining 48 (6.8%) were regarded as patients infected with CagA-negative strains (Table 1). Our histological analysis showed that CagA-positive patients had higher activity and inflammation levels than did the CagA-negative patients, in both the antrum and corpus (*p* < 0.05; Fig 5). Only in the corpus did CagA-positive patients have higher atrophy than did the CagA-negative patients (*p* = 0.001). The number of patients infected with CagA-negative strains was 17.9% (5/28) in the South region, which was higher than that in the Central region (9.4%; 12/128; *p* = 0.19), and significantly higher than that in the North (4.9%, 11/226, *p* = 0.008) and Northeast regions (6.1%, 20/328, *p* = 0.02).

From the 662 patients showing immunoreactivity to the anti-CagA antibody, only 77 (11.6%) were immunoreactive for the α-EAS Ab. Those negative for the α-EAS Ab were regarded as being infected with non-East-Asian type CagA *H*. *pylori*. However, there were no differences in the immunoreactive rates for α-EAS Ab between the four regions (*p* > 0.05; Table 1). We also found no difference in histological scores between patients infected with the East-Asian type CagA *H*. *pylori* and those infected with the non-East-Asian type CagA *H*. *pylori*.

## Discussion

This is the first study to combine histochemical stains and IHC to confirm *H*. *pylori* infections in a large number of samples obtained from four geographical regions, including 17 provinces, in Thailand. This study also analyzed the influence of *H*. *pylori* CagA diversity on gastric mucosal status in Thailand. We revealed that the overall infection rate of *H*. *pylori* was 45.9%, consistent with previous reports (48.2%) [22]. In agreement with the fact that *H*. *pylori* infections occur earlier in life and with a higher frequency in developing countries [23], the prevalence we observed was 43.5% in young patients (aged ≤29 years). This was a lower prevalence than was observed in a high GC risk country, Bhutan, which had a prevalence of 80.4% among young people [24].

Our study confirmed that besides environmental factors, the variation in the ASR of GC in Thailand partly could be explained by *H*. *pylori* infection rate. The Northeast, North, and Central regions had a significantly higher risk for *H*. *pylori* infection than did the South region. The difference in *H*. *pylori* infection rates between regions could be explained by economic and ethnogeographic status in Thailand. With the exception of Bangkok, the total monthly income per household in 2013 in the South region (27,504 THB; approximately 848.6 USD in April 2015) was greater than those in the Central (26,114 THB), North (19,267 THB), and Northeast regions (19,181 THB) (Statistical yearbook Thailand 2013, http://web.nso.go.th). In addition, we also should consider that the South region of Thailand borders Malaysia, and predominantly is ethnically Malayo-Polynesian. Recent reports showed that several seroepidemiological studies in Malaysia consistently found a lower prevalence of *H*. *pylori* amongst Malays (10–25%) compared to the Chinese (35–55%) and Indian (50–60%) populations [25]. On the other hand, most of province included in this study, especially the North and Northeast regions, are predominantly ethnic Thai or Thai-Chinese. Further ethnogeographic studies are necessary to better elucidate the reason for the difference observed in *H*. *pylori* prevalence in Thailand.

In contrast with *H*. *pylori* infection rates, the atrophy score according to the OLGA system was lower in Thailand and Myanmar than in Bhutan (Table 3). All histological results showed that atrophy in both the antrum and corpus was significantly higher in patients positive for *H*. *pylori* than in negative patients. In addition, we observed no difference in gastritis status between regions after adjusting for *H*. *pylori* infection. Moreover, intensity of *H*. *pylori* colonization was correlated with all histological scores in both the antrum and corpus. These results suggest that *H*. *pylori* infection is the most important factor for gastritis risk in every region, and patients infected with *H*. *pylori* can be a high-risk population. Therefore, we suggest that *H*. *pylori* should be eradicated in Thailand.

**Table 3:** Comparison OLGA system in Thailand, Myanmar and Bhutan.

Countries (Ref)	Stage 0	Stage I	Stage II	Stage III	Stage IV
Thailand	34.0%	55.7%	8.6%	1.5%	0.2%
Myanmar[26]	43.2%	52.4%	4.0%	0.4%	0.0%
Bhutan[27]	7.8%	59.0%	27.5%	4.9%	0.8%

Interestingly, in concordance with the prevalence of *H*. *pylori* infection, we found that all histological scores were significantly lower in the South region than in other regions. Moreover, the South region had a lower risk for gastritis, especially corpus-predominant, than other regions. Although only 6.8% of subjects were CagA-negative in this study, the prevalence of CagA-negative strains in the South region was significantly higher than other regions. This suggests that, in addition to host and environmental factors, the low incidence of GC in the South region might be associated with the lower prevalence of *H*. *pylori* infection, precancerous lesions, and CagA-positivity. Our previous meta-analysis found that CagA seropositivity was associated with GC compared with gastritis, even in East Asian countries, although the OR in East Asian countries was less than that of the meta-analysis that included Western countries [28]. Overall, the prevalence of CagA in Thailand is almost similar to that of Vietnam and Myanmar, but lower than that of Bhutan (93.2% vs. 95.1%, 88.4% and 100.0%, respectively) [29]. However, the ASR of GC in Thailand is much lower than that of the three other countries (3.8 vs. 23.7, 15.3, and 23.0, respectively; GLOBOCAN2012). CagA-positivity alone does not reflect the diversity of GC prevalence in each country of this region.

These differences could be attributed to the fact that most Thai subjects were infected with non-East-Asian type CagA *H*. *pylori*, which had a low immunoreactivity for the α-EAS Ab in this study. These results confirmed a study by Chomvarin et al. that also found only 5.8% of *H*. *pylori* in Thailand were East-Asian type CagA [30]. *H*. *pylori* in Vietnam and Bhutan are predominantly East-Asian type CagA, and these countries have a higher GC prevalence than Thailand [31]. Hatakeyama *et al*. described the CM sequence which mediates the interaction of CagA with the kinase catalytic domain of the PAR1/MARK family of serine/threonine kinases are substantially different between Western CM (W-CM) and East Asian CM (E-CM). The fact is E-CM binds more strongly to PAR1 than does W-CM [32]. Additionally, transgenic mice expressing East-Asian-type CagA develop gastrointestinal and hematopoietic malignancies more frequently than those expressing Western-type CagA [33]. However, further studies are needed to explain why East-Asian type CagA *H*. *pylori* are not more virulent than non-East-Asian type CagA *H*. *pylori* in Thailand. Our previous study found that recombination due to transition from East Asian to South Asian genotypes of *H*. *pylori* was frequent in ethnic Thai (42%) and Thai-Chinese (30%), but not in ethnic Chinese [34]. This recombination process could affect protein expression of a large number of genes [35]. A previous report showed Amerindian CagA *H*. *pylori*, which is a hybrid of EPIYA motifs between Western type and East-Asian type, appeared less virulent than both Western and East Asian type CagA *H*. *pylori* [36]. We also should consider the influence of non-*cagA* virulence factors of *H*. *pylori*.

A limitation of this study is that there was no information on ethnic groups. Thailand is a multiethnic country. Further investigation that includes the evaluation of minor ethnic groups is necessary to elucidate the reasons for the low incidence of GC in Thailand. Another limitation is that the α-EAS Ab used in this study was developed in Japan with Japanese-specific strains. It is important to develop an α-EAS Ab using *H*. *pylori* strains that are native to Thailand.

## Conclusion

This is the first study that combined histochemical stains and IHC to confirm *H*. *pylori* infections in a large number of samples obtained from several regions in Thailand. The prevalence of East-Asian type CagA *H*. *pylori* is low in the Thailand population. The low incidence rate of GC in Thailand may be attributed to the low prevalence of precancerous lesions. In addition to host and environmental factors, the low incidence of GC in the South region might be associated with the lower prevalence of *H*. *pylori* infection, precancerous lesions, and CagA-positive *H*. *pylori*.
